# Professional isolation and perceived quality of work life in flexible-employment settings: a cross-sectional study

**DOI:** 10.3389/fpsyg.2026.1965599

**Published:** 2026-10-07

**Authors:** Xingqiang Xu, Rungang Liu

**Affiliations:** 1 Qu Qiubai School of Government, Changzhou University, Changzhou, China; 2 School of Business, Changzhou University, Changzhou, China

**Keywords:** cross-sectional study, flexible employment, occupational wellbeing, perceived career-development constraints, professional isolation, quality of work life

## Abstract

**Introduction:**

This study examines whether professional-isolation scores are associated with perceived quality of work life (QWL) beyond an archived social-isolation index in workers recruited from flexible-employment settings. Perceived career-development constraints are examined as a secondary concurrent correlate.

**Methods:**

Retained questionnaire responses from 237 workers recruited in China were reanalyzed using ordinal measurement assessment, covariate-adjusted composite-score regression, and 5,000 case-resampling bootstrap replications.

**Results:**

Higher professional-isolation scores were associated with lower QWL scores after adjustment for the retained five-item social-isolation index and demographic characteristics (b = −0.307, 95% CI [-0.430, −0.185]). A negative statistical indirect association involving perceived career-development constraints was also observed. Estimates were similar under record-matching and response-pattern sensitivity checks.

**Discussion:**

The study identifies a work-related score association not fully accounted for by the retained social-isolation index. This finding supports examining professional guidance and development opportunities in occupation-specific research. Poor measurement fit and incomplete occupational and eligibility records restrict interpretation to observed-score associations; composite regression establishes neither construct validity nor causal mediation.

## 1 Introduction

Workers can communicate frequently with customers or online communities while lacking people who offer professional guidance, recognition, or information about future opportunities. This problem is relevant outside conventional employment relationships, where career support may be fragmented (Ashford et al., 2018; Idowu and Elbanna, 2022; Petriglieri et al., 2019). Autonomy and access to income can coexist with insecurity, algorithmic control, and uneven well being (Bajwa et al., 2018; Cheung et al., 2025; Wood et al., 2019). This study examines whether professional isolation is associated with perceived quality of work life (QWL) among workers recruited from flexible-employment settings in China.

Professional-isolation research has mainly examined teleworkers who retain organizational ties (Cooper and Kurland, 2002; Golden et al., 2008). Reviews emphasize organizational support and work design (Allen et al., 2015; Bailey and Kurland, 2002; Charalampous et al., 2019; Gajendran and Harrison, 2007), while empirical studies highlight support, voluntariness, and ways to manage isolation (Bentley et al., 2016; Deschênes, 2023; Kaluza and van Dick, 2023; Spilker and Breaugh, 2021). It remains unclear whether similar score associations occur in non-standard-work recruitment contexts. Measurement equivalence across employment arrangements cannot be assumed.

We examine professional isolation and QWL after adjustment for the retained five-item social-isolation index and demographic characteristics. Separate hypotheses address professional isolation, perceived career-development constraints, and QWL; the concurrent indirect association is secondary, and dimensional exploration is *post hoc*. The career-development index concerns perceived restrictions on promotion and development, not emotional strain. The study reports score-level associations alongside the measurement and archival limitations.

### 1.1 Construct scope and theoretical background

Flexible employment includes arrangements differing in contracts, organizational ties, and labor protection, such as platform work, crowdsourcing, self-employment, agency work, and part-time work (Duggan et al., 2020; Tu and Wang, 2024; Vallas and Schor, 2020). Workers' motivations and expectations also differ (Dunn, 2020; Švagan, 2023). Here, flexible employment describes the recruitment settings; respondent-level employment classifications were not verified.

Professional isolation concerns perceived separation from people and resources relevant to work and development. It overlaps with social isolation and loneliness, but contact outside work need not provide professional feedback or recognition (Golden et al., 2008; Marshall et al., 2007; Wright and Silard, 2021). QWL concerns evaluations of working life, with instruments emphasizing different combinations of needs, support, rewards, participation, and well being (Gaucher and Veenhoven, 2022; Martel and Dupuis, 2006; Sirgy et al., 2001; Van Laar et al., 2007). The retained QWL instrument primarily assesses perceived organizational conditions. Clinical health and objective job quality fall outside its scope.

### 1.2 Professional isolation and perceived QWL: H1

Conservation of resources (COR) theory proposes that people seek to obtain and protect valued resources and may experience strain when resources are lost or threatened (Hobfoll, 1989; Halbesleben et al., 2014). Job demands-resources theory likewise identifies feedback, support, autonomy, and development opportunities as resources relevant to motivation and well being (Bakker and Demerouti, 2017). Professional relationships can provide information about performance, recognition of contributions, and opportunities to learn. Limited access to such relationships may therefore accompany less favorable evaluations of work. This argument concerns the availability and usefulness of professional support, not simply the frequency of contact (Van Zoonen and Sivunen, 2022; Wang et al., 2021).

1. *Hypothesis 1. Higher professional-isolation scores are associated with lower QWL scores after adjustment for the retained five-item social-isolation index and demographic characteristics*.

### 1.3 Professional isolation and perceived career-development constraints: H2

Professional contact can provide career resources. Informal learning, networking, and mentoring support development (Cooper and Kurland, 2002), and the career resources model identifies social resources as relevant to career management (Hirschi, 2012). Limited guidance and professional access may therefore accompany greater perceived development constraints. This expectation is relevant where workers construct careers without conventional promotion ladders (Idowu and Elbanna, 2022; Petriglieri et al., 2019). Research on algorithmic management describes both work demands and resources (Duggan et al., 2020; Kadolkar et al., 2025; Lang et al., 2023), but algorithmic management was not measured here. These arguments motivate the association hypothesis; they do not validate the retained two-item index.

1. *Hypothesis 2. Professional-isolation scores are positively associated with perceived career-development constraints scores*.

### 1.4 Perceived career-development constraints and perceived QWL: H3

Opportunities for advancement and development are relevant to QWL because they affect what workers believe their present work can offer them. Sirgy et al. (2001) locate QWL in the satisfaction of several needs, including actualization and knowledge: work can provide opportunities to realize professional potential and develop skills. Perceiving little room for advancement or development may therefore accompany a less favorable assessment of working life, even when current tasks remain manageable. Gaucher and Veenhoven (2022) likewise identify self-development among the aspects covered by QWL questionnaires, although they show that the instruments differ substantially in content. This conceptual link is not identical to professional isolation. Professional disconnection concerns access to work-related contact and resources, whereas the two retained constraints items concern respondents' assessments of their development prospects. These assessments may relate to QWL beyond the association captured by professional-isolation scores. We therefore expect a negative concurrent association between perceived career-development constraints and QWL after adjustment for PI. This rationale does not validate the two-item index or establish that development constraints cause lower QWL.

1. *Hypothesis 3. Perceived career-development constraints scores are negatively associated with QWL scores after adjustment for professional-isolation scores*.

### 1.5 Secondary concurrent indirect analysis

H2 and H3 motivate examining the product of the professional-isolation-to-constraints and conditional constraints-to-QWL coefficients. All variables were measured concurrently, however, and such products need not represent longitudinal processes (Maxwell and Cole, 2007; Maxwell et al., 2011). We therefore examine the indirect association as a secondary statistical decomposition. It has no separate confirmatory hypothesis and does not test causal mediation. Bootstrap intervals quantify uncertainty in the coefficient product; they cannot establish temporal ordering.

### 1.6 *Post-hoc* dimensional exploration: RQ1

The six professional-isolation items include development-related access and collegial connection. Professional-development and workplace-isolation research makes that distinction plausible (Cooper and Kurland, 2002; Marshall et al., 2007), but does not validate the proposed two- and four-item subsets. We therefore retain the following question as *post hoc*.

1. *Exploratory research question 1. What dimensional patterns are suggested by the six professional-isolation items, and how do the previously proposed developmental-resource and relational scores relate to perceived career-development constraints and QWL when considered together?*

H1-H3 are theory-guided association hypotheses; the indirect product is secondary and RQ1 is exploratory. No preregistration record was available, and the archive does not establish prospective specification before original data collection. The candidate CFA and revision-stage EFA use the same respondents and provide no independent validation.

## 2 Materials and methods

### 2.1 Design and participants

This cross-sectional reanalysis used retained item-level questionnaire records. Reporting follows relevant STROBE guidance (von Elm et al., 2007). Gaps in the archived records are identified below.

Non-probability, setting-based recruitment targeted flexible or non-standard workers in China, including online shop owners, micro-entrepreneurs, real-estate agents, ride-hailing drivers, delivery workers, crowdsourcing workers, and other flexible or part-time workers. Paper questionnaires were distributed at delivery and pickup locations; some online shop owners and crowdsourcing workers were approached through WeChat groups. Exact collection dates, a separate minimum-age eligibility item, and respondent-level occupational-screening records were not preserved. The analytic file also lacks occupation, contract type, platform dependence, work location, recruitment channel, and identifiers for specific recruitment locations or WeChat groups. These gaps prevent independent verification of eligibility and reporting of occupation-specific proportions. The sample cannot be treated as representative of gig workers or any single employment category.

The retained study account reports 248 returned questionnaires, with 11 excluded for missing data or suspected careless responding, leaving 237 records. The excluded questionnaires and case-level reasons were not retained. All focal items and five demographic variables are complete in the retained file. The subsequent record-matching and response-pattern checks were separate sensitivity analyses, not a reconstruction of the original exclusions.

No a priori sample-size calculation was documented. We analyzed all 237 retained records and emphasize interval estimates and sensitivity checks, not retrospective power. The sample size alone does not establish adequacy for multi-factor measurement assessment; no occupation-specific or measurement-invariance analyses were attempted.

### 2.2 Procedure and ethical considerations

Informed-consent information was incorporated into the questionnaire used for the survey. Participants indicated their consent through a questionnaire-embedded checkbox or confirmation item stating that they had read the information and agreed to participate. Individuals who did not wish to participate or considered that their circumstances did not meet the survey requirements could decline or discontinue participation. The retained questionnaire introduction describes the study's academic purpose, anonymity, and confidentiality. The separate ethics certificate records voluntary informed consent before participation.

The study was reviewed and approved by the Institutional Review Board/Ethics Committee of the Qu Qiubai School of Government, Changzhou University (Protocol No. CZU-SG-2025-010; decision dated 25 May 2025). Data collection took place after this approval, as confirmed by the authors. The exact data-collection dates were not retained in the archived study records.

### 2.3 Measures

Scores were recalculated from retained item responses; no responses were generated or imputed. The current questionnaire lists A = very consistent through E = very inconsistent; SAV labels map these anchors to 1–5. Each included item was transformed as 6—x and averaged. Higher scores denote professional isolation, perceived career-development constraints, endorsement of the retained social-isolation items, or favorable QWL, as appropriate. Supplementary Table S1 provides the exact item sets. The current questionnaire's file hash differs from that recorded for an earlier version. This difference does not show whether the wording changed; equivalence to the administered version remains unverified. The saved composites were untransformed item means. Simultaneously reversing the focal scales preserves the principal linear slopes and product ab algebraically; this checks the coding but provides no independent validation.

The focal models used 26 of the 34 research items: six professional-isolation items, two career-development constraints items, five social-isolation items, and 13 QWL items. Four relationship/work-support items (rs1–rs4) and the other four social-isolation items (si1, si3, si7, and si8) were not included. The rs block covers conflict with colleagues, lack of supervisory support or help, and feeling isolated at work. The focal models retain the previously analyzed item sets; they do not model every item in the questionnaire. The original item-selection and adaptation decisions, including the historical basis for omitting the rs block and selecting five SI items, could not be independently reconstructed. No items were added or removed in this reporting revision on the basis of their associations or fit. Supplementary Section S1 and the accompanying variable codebook document the included and excluded fields.

#### 2.3.1 Professional isolation

The six-item professional-isolation composite covers missed development activities, lack of guidance, exclusion from professional circles, limited face-to-face contact, little collegial emotional support, and limited informal interaction (α = 0.854). An example is, “I miss out on many activities and meetings that could advance my career.” Documentation links the items to professional-isolation research (Cooper and Kurland, 2002; Golden et al., 2008), but full adaptation and pilot-testing records are unavailable. The pi1-pi2 developmental-resource and pi3-pi6 relational subsets are examined only in *post-hoc* analyses.

#### 2.3.2 Perceived career-development constraints

Two items assessed slow promotion or no promotion prospects and insufficient personal development space. Their recoded mean had α = 0.834. We use the label perceived career-development constraints to reflect this content; the items do not directly assess emotional or physiological stress responses. Occupational-stress and promotion-stress research provides related conceptual context (Carlson and Rotondo, 2001; Cooper and Marshall, 1976), not validation of this exact item combination. Because item-selection and adaptation records are incomplete, we interpret the score as a narrow observed index. The revised label does not change its items, scoring, or estimates; legacy code identifiers are explained in Supplementary Section S1.

#### 2.3.3 Perceived quality of work life

The 13-item QWL mean covers organizational assistance, evaluation, rewards, recognition, relationships, fairness, decision disclosure, and organizational attractiveness (α = 0.840; Supplementary Table S1). One item states, “The pay and benefits in my organization are above the industry average.” The item qwl7 refers to disclosure of decisions affecting employees' interests, not participation in decision-making. Higher recoded scores indicate more favorable evaluations.

Documentation attributes the items to Qing et al. (2010), but the original-to-adapted mapping and scoring protocol were not independently recovered. Several items assume an identifiable organization or supervisor and may differ in meaning across work arrangements. We retained the previously analyzed item set rather than selecting a new outcome for favorable fit. Its mean is treated as an observed work-life evaluation, not a validated unidimensional latent construct.

#### 2.3.4 Social-isolation adjustment index

The saved social-isolation composite used si2, si4, si5, si6, and si9 from nine available items. These address loneliness, distrust, unreliable relationships, social caution, and the belief that most married people in China have unsatisfactory lives. The last item is not a direct personal-isolation measure. We retained this set after recoding (α = 0.825); its selection rationale and exact source were not recovered. The earlier documentation cites McTavish et al. (1997), a study of content analysis that does not establish the provenance of these Likert items. Analyses omitting this archival adjustment index are also reported. Adjustment for this retained five-item social-isolation index is not equivalent to fully accounting for the broader social-isolation construct.

#### 2.3.5 Demographic covariates

The categorical covariates were sex, education, age group, work tenure, and job level. Education was collapsed to associate degree or lower vs. bachelor's degree or higher because only one participant had a master's degree or higher. Job level was collapsed to employee, first-line manager, and middle/senior manager because only four were senior managers. Adjustment is descriptive and does not establish a sufficient causal adjustment set.

### 2.4 Measurement evaluation

Cronbach's α describes internal consistency, not unidimensionality (McNeish, 2018). Ordinal CFA was fitted in lavaan using WLSMV estimation, ordered indicators, theta parameterization, unit latent variances, and the nlminb optimizer. WLSMV uses diagonally weighted least squares for point estimates with robust standard errors and an adjusted test statistic. All retained variables were complete, so listwise handling excluded no cases. Neither FIML nor imputation was used. The complete focal four-factor and five-factor candidates used the same 26 items; the latter divided professional isolation into its *post-hoc* subsets. No latent structural regression was fitted.

We report scaled WLSMV χ^2^, robust categorical CFI/TLI and RMSEA with 90% intervals, and SRMR (Savalei, 2021). Standard/scaled alternatives remain in the supplement, without replacing unfavorable robust indices. CR and AVE use fully standardized underlying-response loadings; HTMT2 uses absolute polychoric correlations with geometric-mean aggregation (Henseler et al., 2015; Roemer et al., 2021). Formulas and values appear in Tables 1, 2; loadings, residuals, and alternative fits are supplementary. These diagnostics are model-dependent and cannot override global misfit.

**Table 1:** Internal consistency and model-dependent reliability/variance summaries.

Candidate	Score/factor	Items	α	CR	AVE	Loading range
Four-factor	Professional isolation	6	0.854	0.900	0.600	0.690–0.815
Four-factor	Perceived career-development constraints	2	0.834	0.884	0.794	0.804–0.970
Four-factor	Social-isolation index	5	0.825	0.859	0.553	0.600–0.842
Four-factor	QWL	13	0.840	0.869	0.351	0.398–0.858
Five-factor	Developmental-resource subset	2	0.689	0.743	0.591	0.754–0.783
Five-factor	Relational subset	4	0.861	0.894	0.680	0.734–0.867
Five-factor	Perceived career-development constraints	2	0.834	0.885	0.795	0.802–0.972
Five-factor	Social-isolation index	5	0.825	0.860	0.553	0.602–0.842
Five-factor	QWL	13	0.840	0.869	0.351	0.398–0.857

CR and AVE use standardized underlying-response loadings. CR = (Σλ)^2^/[(Σλ)^2^ + Σ(1 – λ^2^)]; AVE = Σλ^2^/k. These values describe the fitted candidates and do not negate their inadequate global fit. α is computed from the observed recoded items.

**Table 2:** HTMT2 for the five-factor candidate.

Factor	1	2	3	4	5
1. Developmental-resource subset	—	—	—	—	—
2. Relational subset	0.713	—	—	—	—
3. Perceived career-development constraints	0.742	0.463	—	—	—
4. Social-isolation index	0.805	0.612	0.489	—	—
5. QWL	0.602	0.351	0.553	0.395	—

Absolute polychoric correlations; geometric-mean aggregation. Lower triangle shown; *N* = 237. Low ratios alone do not validate this poorly fitting candidate.

Two-indicator factors were embedded in correlated simple-structure models with free loadings and independent item residuals. Equal loadings were not imposed. Full-rank local derivative diagnostics and available standard errors supported local numerical identification. They do not, however, establish adequate content coverage or stability. This caution applies to both the developmental-resource subset and the career-development index (Supplementary Section S2).

### 2.5 Response coding and data-quality audit

An out-of-range value in an earlier CSV (pi2 = 43) was corrected to 3 following author confirmation. The reported analyses used the retained SAV dataset, which was not modified; a corrected CSV was regenerated from it. Correction provenance and limitations of retrospective verification are documented in the Supplementary Material.

Eight pairs of records matched exactly on all five demographic variables and all 34 items. Without respondent identifiers, timestamps, or channel markers, they cannot be classified conclusively as duplicate submissions, duplicate entry, or coincidentally identical responses. Retaining one record per pair yielded *n* = 229. Removing three further long-string records (one constant across all items; two with at least 23 consecutive identical answers) yielded *n* = 226. The primary analysis retained all 237 records. The reduced samples test sensitivity to these response patterns; they do not establish respondent independence.

### 2.6 Statistical analysis

For H1, QWL was regressed on mean-centered professional isolation and the retained five-item social-isolation index plus the categorical covariates. We used ordinary least squares with heteroskedasticity-consistent HC3 standard errors and 95% confidence intervals. We report unstandardized b, standardized β, R^2^, adjusted R^2^, incremental R^2^, and f^2^. The PI coefficient c is the adjusted association before conditioning on perceived career-development constraints, not a causal direct effect.

For H2, perceived career-development constraints were regressed on PI and the same covariates (a). For H3, QWL was regressed on PI, perceived career-development constraints, and the covariates, yielding b and the conditional PI coefficient c′. For the secondary product ab, we used 5,000 case-resampling bootstrap draws (seed 20260713) and percentile 95% intervals. The completely standardized product, ab_std = ab × SD(PI)/SD(QWL), was recalculated with both regressions and marginal SDs in every draw, not by rescaling interval endpoints (Cheung, 2009). Component intervals use HC3; product intervals use bootstrap.

The resampling unit was the individual retained record. Recruitment-location and WeChat-group identifiers were not retained, so dependence within recruitment sources could not be assessed or modeled. HC3 and record-level bootstrap intervals do not adjust for such clustering.

Analyses were repeated at *n* = 237, 229, and 226. Additional checks omitted the retained five-item social-isolation index, added a quadratic PI term, fitted Huber regression, and examined variance-inflation factors and Cook's distances. Tests were two-sided, with *p* < 0.05 as a conventional reference. Composite regression estimates score-level associations; it does not repair poor measurement fit or remove measurement error (Cole and Preacher, 2014).

To address RQ1, we combined the earlier *post-hoc* candidate CFA with revision-stage polychoric EFA using MINRES, oblimin rotation, and 95th-percentile parallel analysis (Fabrigar et al., 1999; Supplementary Section S4). No items were dropped to improve fit. The previously proposed two- and four-item scores were also entered jointly in exploratory regressions. Direct coefficient contrasts were estimated rather than inferring differences from separate significance tests (Gelman and Stern, 2006). All dimensional analyses use the same retained sample and are hypothesis-generating.

Python was used for deterministic scoring, OLS/HC3, and bootstrap; R was used for ordinal CFA (lavaan), HTMT2 (semTools), and factor analysis (psych). The analyses conducted for this revision use the retained study records and do not constitute a new dataset or independent replication. Exact software versions, seeds, input/output hashes, and run instructions are in Supplementary Section S6 and the reproducibility package.

## 3 Results

### 3.1 Participant characteristics and data quality

The sample included 113 women (47.7%) and 124 men (52.3%); 63.7% were labeled in management categories, including 53.6% first-line managers (Table 3). These labels do not verify employment type. The youngest age category is labeled 18–25 years in the questionnaire but below 25 in the SAV. Neither label establishes a separate minimum-age screening process. All retained items and covariates were complete in the full and sensitivity samples.

**Table 3:** Retained participant characteristics (*N* = 237).

Characteristic	Category	*n*	%
Sex	Woman	113	47.7
	Man	124	52.3
Education	Associate degree or lower	122	51.5
	Bachelor's degree	114	48.1
	Master's degree or higher	1	0.4
Age	Age category 1^*^	21	8.9
	26–35 years	83	35.0
	36–45 years	89	37.6
	Older than 45 years	44	18.6
Work tenure	Less than 3 years	28	11.8
	3–5 years	13	5.5
	6–10 years	62	26.2
	More than 10 years	134	56.5
Job level	Employee	86	36.3
	First-line manager	127	53.6
	Middle manager	20	8.4
	Senior manager	4	1.7

^*^The questionnaire labels the first age category 18–25 years, whereas the SAV label states below 25 years. This category is not proof of minimum-age screening. Job-level labels do not establish occupation or contract type.

### 3.2 Descriptive and measurement results

Descriptive statistics and correlations appear in Table 4. PI correlated positively with perceived career-development constraints (r = 0.499) and the retained five-item social-isolation index (r = 0.619), and negatively with QWL (r = −0.416). Perceived career-development constraints correlated with QWL at r = −0.459. These score correlations do not establish discriminant validity.

**Table 4:** Descriptive statistics and Pearson correlations.

Score	*M*	SD	α	1	2	3	4
Professional isolation	2.57	0.72	0.854	1.000	—	—	—
Perceived career-development constraints	3.23	1.01	0.834	0.499	1.000	—	—
Social-isolation index	3.07	0.72	0.825	0.619	0.401	1.000	—
QWL	2.86	0.56	0.840	−0.416	−0.459	−0.354	1.000

All scores are item means after 6 – x recoding; *N* = 237. The diagonal is 1.000. Reliability and correlations do not establish unidimensionality.

Both complete measurement models fitted poorly (Table 5). The five-factor model had robust CFI = 0.775, TLI = 0.747, RMSEA = 0.108, and SRMR = 0.075; four-factor fit was poorer. Five-factor robust CFI remained below 0.800 in both reduced samples (Supplementary Table S5), so response-quality screening did not resolve misfit.

**Table 5:** Complete ordinal measurement-model fit (*N* = 237).

Candidate	Scaled χ^2^ (df)	Robust CFI	Robust TLI	Robust RMSEA	RMSEA 90% CI	SRMR
Four-factor	801.09 (293)	0.728	0.698	0.118	[0.111, 0.125]	0.083
Five-factor	693.72 (289)	0.775	0.747	0.108	[0.101, 0.115]	0.075

WLSMV, ordered indicators, theta parameterization. Four-factor: PI, perceived career-development constraints, the retained five-item social-isolation index, QWL. Five-factor: PI is split into two *post-hoc* subsets. Both models use the same 26 items and fit inadequately. Standard and scaled alternatives appear in Supplementary Table S11.

QWL AVE was 0.351, with six standardized loadings below 0.500 (Table 1). Five-factor HTMT2 ratios ranged from 0.351 to 0.805 (Table 2), but do not override inadequate global fit. Fit statistics for the local PI candidates and the full item-level diagnostics are reported in the supplement. No candidate is interpreted as a validated latent structural model.

### 3.3 Primary association hypotheses: H1-H3

PI was associated with lower QWL after adjustment, b = −0.307, 95% HC3 CI [−0.430, −0.185], β = −0.396, consistent with H1. The model explained 30.0% of QWL variance (adjusted R^2^ = 0.263). PI added 0.086 to explained variance, with f^2^ = 0.123 (Table 6).

**Table 6:** Primary and component observed-score regression associations (*N* = 237).

Association	*b*	HC3 SE	95% HC3 CI	β	*p*	R^2^
PI–QWL (c)	−0.307	0.062	[−0.430, −0.185]	−0.396	< 0.001	0.300
PI–constraints (a)	0.614	0.110	[0.398, 0.830]	0.437	< 0.001	0.312
Constraints–QWL (b)	−0.163	0.038	[−0.238, −0.088]	−0.295	< 0.001	0.360
PI–QWL, conditional (c′)	−0.207	0.061	[−0.326, −0.089]	−0.267	< 0.001	0.360

All models adjust for the retained five-item social-isolation index and categorical sex, education, age group, tenure and job level. The QWL model yielding b and c′ additionally includes perceived career-development constraints; c does not. β uses marginal observed-score SDs. Labels denote regression components, not identified causal effects. The label 'constraints' denotes perceived career-development constraints.

PI was positively associated with perceived career-development constraints (a = 0.614), and perceived career-development constraints were negatively associated with QWL conditional on PI and covariates (b = −0.163), consistent with H2 and H3. The conditional PI coefficient was c′ = −0.207. Table 6 reports HC3 intervals and standardized coefficients for each component.

### 3.4 Secondary indirect association involving perceived career-development constraints

The product ab was −0.100, with a 95% percentile bootstrap interval [−0.169, −0.045]; the completely standardized product was −0.129, with interval [−0.210, −0.062]. All 5,000 draws were estimable (Table 7). These are non-zero concurrent statistical associations, not evidence that PI preceded perceived constraints or that those constraints transmitted a causal effect.

**Table 7:** Unstandardized and completely standardized statistical indirect associations.

Sample (n)	Quantity	Estimate	Bootstrap SE	95% bootstrap CI
Full (237)	ab	−0.100	0.031	[−0.169, −0.045]
Full (237)	ab_std	−0.129	0.038	[−0.210, −0.062]
Record-reduced (229)	ab	−0.094	0.030	[−0.159, −0.042]
Record-reduced (229)	ab_std	−0.121	0.038	[−0.202, −0.055]
Pattern-screened (226)	ab	−0.093	0.031	[−0.160, −0.040]
Pattern-screened (226)	ab_std	−0.120	0.038	[−0.202, −0.052]

5,000 record-resampling draws for each sample; seed 20260713; percentile intervals; zero rank-deficient draws. ab_std = ab × SD(PI)/SD(QWL), recomputed within each draw. These are concurrent statistical associations, not causal mediation effects.

### 3.5 Robustness analyses

The adjusted PI-QWL association remained negative in the record-reduced sample, b = −0.303, 95% CI [−0.430, −0.177], and the pattern-screened sample, b = −0.314, 95% CI [−0.441, −0.188]. Table 7 reports the unstandardized and completely standardized indirect products for each sample; Supplementary Table S6 reports regression sensitivity results. Similar estimates do not establish response independence or measurement validity.

Omitting the retained five-item social-isolation index yielded b = −0.381 (*p* < 0.001); Huber regression yielded b = −0.274. The quadratic PI term was not supported (b = 0.056, *p* = 0.221). PI and SI variance-inflation factors were 1.82 and 1.75; 13 observations exceeded Cook's-distance threshold 4/n. These checks do not support a curvilinear association or rule out bias from influential responses.

### 3.6 *Post-hoc* dimensional exploration

The PI two-factor candidate improved local fit, but its robust RMSEA 90% interval was wide, [0.000, 0.120]. Common-factor parallel analysis suggested two factors, whereas the component reference suggested one component. In the two-factor oblimin solution, pi1 had loading 1.001 and uniqueness 0.003; pi2 cross-loaded 0.442/0.370. The loadings did not clearly recover the proposed two-/four-item split (Supplementary Section S4). Same-sample EFA and CFA therefore do not independently validate either subset.

Exploratory regressions and direct contrasts are reported in Supplementary Tables S8–S9. The difference between the two adjusted QWL coefficients was −0.121, 95% bootstrap CI [−0.328, 0.078]; the indirect-product difference was −0.059, CI [−0.124, −0.013]. Thus, evidence differs across the specific contrasts and does not establish generally distinct dimensional roles. These *post-hoc* results are not used to redefine H1-H3 or replace the primary six-item PI score.

## 4 Discussion

### 4.1 Principal findings and contribution

Higher professional-isolation scores were associated with poorer perceived QWL after adjustment for the retained five-item social-isolation index and demographic characteristics. PI added 0.086 to explained variance, and estimates were similar across record-matching, response-pattern, and robust-regression checks. The secondary analysis identified a concurrent indirect association involving perceived career-development constraints. Within this recruitment sample, professional-isolation scores contributed information about QWL scores beyond the retained social-isolation index and demographic categories. The finding supports studying work-related disconnection alongside these broader social appraisals, with interpretation confined to the specified scores.

### 4.2 Professional isolation as a work-related resource gap

The findings are compatible with COR and job demands-resources accounts: professional contacts can provide feedback, recognition, opportunity information, and learning resources (Hobfoll, 1989; Halbesleben et al., 2014; Bakker and Demerouti, 2017). Professional-isolation scores were related to QWL scores in the retained recruitment sample. However, absent occupation and contract records prevent conclusions about particular flexible-work categories. The association persisted with and without adjustment for the retained five-item social-isolation index. This comparison concerns that recorded index; it does not establish an association independent of the broader social-isolation construct. Measurement overlap and error remain possible.

### 4.3 Perceived career-development constraints as a concurrent indirect correlate

The two career-development items concern promotion prospects and development space. The observed pattern links professional-isolation scores, perceived career-development constraints scores, and work-life evaluations. This association does not demonstrate an emotional stress mechanism. Content overlap among these measures may contribute to the observed associations. Reverse appraisal, reciprocal relationships, shared negative affect, and unmeasured work conditions remain plausible (Maxwell and Cole, 2007; Maxwell et al., 2011). Future longitudinal studies should distinguish resource access, threat appraisal, and emotional strain, while measuring relevant confounders; temporal separation alone would not establish causality.

### 4.4 Exploratory dimensional pattern

Future item development could examine developmental access and collegial connection more closely. The stability of the proposed two-/four-item split has not been established: the EFA second factor is dominated by pi1, whereas pi2 cross-loads (Section 3.6). Local numerical identification and improved PI-only CFA fit do not establish stable dimensions or adequate content coverage. The same respondents supplied both EFA and CFA data, so these analyses provide no independent confirmation and cannot support distinct dimensional mechanisms. Relational isolation should not be declared unimportant because its adjusted interval includes zero, nor should differing significance statuses be treated as evidence of a difference (Gelman and Stern, 2006). Future studies need broader item pools, cognitive interviews, documented adaptation, and independent exploratory and confirmatory samples.

### 4.5 Practical implications

Professional guidance and development opportunities warrant further assessment. Their effectiveness as interventions was not tested in this study. Organizations, platforms, labor-service agencies, and worker communities can examine access to useful feedback, recognition, mentoring, peer consultation, and portable skill information. Increasing message volume or requiring online-group participation may add contact without supplying usable support. Evaluation should distinguish employment types, retain contract and platform information, and assess concrete QWL domains alongside global evaluations.

### 4.6 Measurement implications

Measurement validity remains a central unresolved limitation. The complete five-factor model's robust CFI = 0.775 and RMSEA = 0.108 indicate poor global fit; QWL AVE = 0.351 and six loadings below 0.500 provide additional evidence of weak measurement (Tables 5, 1). The five-factor model also leaves 66 of 325 item-pair absolute correlation residuals above 0.100 (Supplementary Table S4). These concerns are not resolved by favorable local PI fit, internal consistency, or HTMT2 ratios. Using composite-score regression permits a limited description of associations among the specified item means; it does not repair the failed measurement model, establish convergent or discriminant validity, or correct measurement error. Uncorrected error can bias multivariable coefficients in either direction (Cole and Preacher, 2014). Organization-centered QWL content may also be interpreted differently across work arrangements. The estimates should therefore not be treated as relationships among validated, equivalent latent constructs. Documented item adaptation and independent measurement validation are needed before making stronger construct-level claims.

### 4.7 Strengths and limitations

Scoring decisions and unfavorable measurement findings are reported explicitly. The study also uses HC3 inference, standardized and unstandardized bootstrap intervals, and separate record-matching and response-pattern sensitivity analyses. These features make the analysis easier to audit, but the following limitations remain.

First, the cross-sectional, single-source design leaves temporal ordering, unmeasured confounding, and common-method processes unresolved. Poor one-factor fit would not rule out common-method bias (Podsakoff et al., 2003).

Second, non-probability recruitment and incomplete occupational and eligibility records leave the composition of the target sample uncertain. Occupation, contract type, platform dependence, and individual screening cannot be reconstructed. The management-category labels in Table 3 cannot substitute for occupational classifications or verify flexible-employment eligibility. Consequently, findings describe the retained recruitment sample, not a verified homogeneous group or a representative population of Chinese flexible workers. Exact collection dates, the original 11 excluded questionnaires and case-level reasons, and a separate minimum-age screening record were not retained; equivalence of the retained questionnaire to the administered version also remains unverified. These gaps cannot be filled by statistical adjustment or revised wording.

Third, without identifiers or timestamps, the causes of eight identical-record pairs and three long-string patterns cannot be established. Possible dependence among respondents recruited through the same pickup location or WeChat group could not be assessed because recruitment-cluster identifiers were not retained. HC3 standard errors and record-level bootstrap intervals do not account for such clustering, leaving the coverage of the reported intervals uncertain if within-source dependence was present. Removing exactly matching response patterns is not a correction for recruitment clustering.

Fourth, incomplete measure provenance, the narrow career-development constraints index, undocumented SI item selection, and inadequate full-model fit constrain construct interpretation as detailed in Section 4.6. Same-sample dimensional exploration has not established a stable split and does not resolve these problems. Finally, *N* = 237 and unverified occupational strata limit dimensional precision and subgroup analysis. Objective network position, earnings stability, workloads, contracts, social protection, performance, and clinical health were not measured. Stronger occupation-specific studies should address these gaps before practical or mechanistic conclusions are drawn.

## 5 Conclusion

In this sample of workers recruited from flexible-employment settings, professional-isolation scores were negatively associated with QWL scores after adjustment for the retained five-item social-isolation index and demographic characteristics. The secondary analysis identified a concurrent association involving perceived career-development constraints. Poor measurement fit, incomplete occupational and eligibility records, and unconfirmed dimensional stability remain unresolved. Composite regression does not remove these limitations. The findings motivate better measurement of professional-resource access; they do not establish causal mediation or validate a dimensional theory. Studies with clearly classified occupations, documented measures, independent measurement validation, and longitudinal designs are needed.

## Data Availability

The raw data supporting the conclusions of this article will be made available by the authors, without undue reservation.
