# Supplementary material for: Professional isolation and perceived quality of work life in flexible-employment settings: a cross-sectional study
Source: Front Psychol. 2026 Oct 7;17:1965599. doi: 10.3389/fpsyg.2026.1965599 (PMC13683001; doi:10.3389/fpsyg.2026.1965599)
Supplement: Supplementary file 1 [file Data_Sheet_1.pdf]

Supplementary material

Professional isolation and perceived quality of work life in flexible-employment settings: A cross-sectional study

Supplementary material accompanying the revised manuscript | 7 September 2026. Statistical estimates are retained from the completed reanalysis; this reporting revision introduces no newly collected data or new estimates. No participant-level source data are included.

S1. Item provenance, scoring and sample accounting

The retained anchors map 1 to very consistent and 5 to very inconsistent. Each of the 26 included item responses is transformed as $6 - x$ before averaging. Higher PI, perceived career-development constraints and social-isolation index scores denote stronger endorsement of their negative content; higher QWL denotes more favorable organizational/work-life evaluations. The saved composites were untransformed item means and were not used as the aligned analysis scores. Simultaneously reversing the focal scales preserves the principal linear slopes and product ab algebraically; this is a coding check, not independent validation. Content summaries below are descriptive English paraphrases, not a newly validated translation. Exact source-to-adaptation mapping remains incomplete. See main Section 2.3.

Table S1. Item sets and coding.

ID	Score	Content summary	Recoding
pi1	PI	Missed career-development activities/meetings	6 − x
pi2	PI	No opportunity for guidance at work	6 − x
pi3	PI	Feeling excluded from circles	6 − x
pi4	PI	Limited face-to-face contact with colleagues	6 − x
pi5	PI	Little emotional support from colleagues	6 − x
pi6	PI	Little private interaction with colleagues	6 − x
cds1	Perceived career-development constraints	Slow promotion or no promotion prospects	6 − x
cds2	Perceived career-development constraints	Insufficient personal development space	6 − x
si2	Social-isolation index	Feeling lonely in the world	6 − x
si4	Social-isolation index	Others are insufficiently sincere	6 − x
si5	Social-isolation index	Belief that most married people in China have unsatisfactory lives	6 − x
si6	Social-isolation index	Fewer dependable interpersonal relationships	6 − x
si9	Social-isolation index	Need for special caution in contemporary society	6 − x
qwl1	QWL	Choice of work partners	6 − x
qwl2	QWL	Organizational performance evaluation	6 − x
qwl3	QWL	Organizational help with work	6 − x
qwl4	QWL	Periodic rewards	6 − x
qwl5	QWL	Praise for excellent employees	6 − x

ID	Score	Content summary	Recoding
qwl6	QWL	Spontaneous member gatherings	6 − x
qwl7	QWL	Disclosure of decisions affecting employees' interests	6 − x
qwl8	QWL	Fair treatment	6 − x
qwl9	QWL	Supervisor thanks after task completion	6 − x
qwl10	QWL	Low employee turnover	6 − x
qwl11	QWL	Many applicants during recruitment	6 − x
qwl12	QWL	Above-industry-average pay/benefits	6 − x
qwl13	QWL	Good organizational reputation	6 − x
rs1–rs4	Excluded from focal models	Relationship/work-support stress items	Not used
si1, si3, si7, si8	Excluded from five-item SI index	Remaining social-distance items; not added post hoc	Not used

Note. PI = mean(pi1–pi6); CDS = mean(cds1, cds2); SI = mean(si2, si4, si5, si6, si9); QWL = mean(qwl1–qwl13), all after transformation. Exploratory developmental-resource subset = mean(pi1, pi2); relational subset = mean(pi3–pi6). Selection history of the five SI items is unavailable; si5 is a broad marital-life judgment, not a direct personal-isolation item. CDS denotes perceived career-development constraints, not a measure of emotional stress.

The 26-item focal set excludes rs1-rs4 and si1, si3, si7, and si8 from the 34 research items. The rs items describe conflict with colleagues, lack of supervisory support, supervisors being unable or unwilling to help, and feeling isolated at work. They remain outside the previously analyzed focal sets. Their wording overlaps with work support and isolation, but does not justify adding them retrospectively to PI. The original selection/adaptation decisions and the historical basis for omitting this block could not be independently reconstructed. The current reporting revision does not change item membership. The accompanying codebook covers every retained SAV field and the variables derived by the archived analysis scripts, including value labels, score direction, covariate recoding, reference categories, and sensitivity-sample rules.

The two-item observed index is referred to as perceived career-development constraints to reflect its item content more precisely. This terminology change does not alter the items, 6 − x recoding, composite calculation, or statistical estimates. Legacy identifiers, including cds1, cds2, cds, cds_c, and Career_Stress, are retained in the archived scripts and outputs for reproducibility and refer to the same index; cds_c is its mean-centered score.

Original retained account: 248 returned → 11 original exclusions → 237 retained. The excluded cases and allocation of reasons were not archived. Separate sensitivity sequence: 237 → 229 after retaining one record from each of eight exactly matching pairs → 226 after removing three long strings (threshold 23 consecutive identical responses across 34 items in questionnaire order). Neither sequence validates individual respondent eligibility or independence.

An out-of-range value in an earlier CSV (pi2 = 43) was corrected to 3 following author confirmation. The reported analyses used the retained SAV dataset, which was not modified; a corrected CSV was regenerated from it. All 34 item values in the retained SAV are within 1–5, and the retained analytic variables have no missing cells. The earlier erroneous CSV was not retained, so its original row-level discrepancy cannot now be independently reconstructed. The retained SAV and regenerated CSV do not by themselves establish the origin of the earlier error. The main text reports the correction in Section 2.5.

Questionnaire-version provenance is a separate issue. The current questionnaire's file hash differs from that recorded in an earlier audit. A file-hash difference does not establish whether wording changed, and equivalence to the version administered to participants remains unverified. The author's confirmation of the consent procedure does not independently resolve questionnaire-version equivalence or the incomplete source-to-adaptation mapping.

S2. Measurement diagnostics

Ordinal CFA: lavaan WLSMV, theta parameterization, ordered indicators, latent variances fixed at 1, nlminb optimizer; listwise setting with no missing records. No equal-loading constraints, residual covariances or post-hoc item deletions were imposed. No latent structural regression was fitted. CR/AVE are calculated on the standardized underlying-response scale and are conditional on the candidate model.

Table S2. Standardized candidate-CFA loadings.

Item	Four-factor	Five-factor	PI-only two-factor
pi1	0.767	0.754	0.682
pi2	0.778	0.783	0.866
pi3	0.813	0.867	0.827
pi4	0.815	0.863	0.898
pi5	0.779	0.827	0.817
pi6	0.690	0.734	0.747
cds1	0.804	0.802	—
cds2	0.970	0.972	—
si2	0.748	0.744	—
si4	0.735	0.737	—
si5	0.773	0.774	—
si6	0.842	0.842	—
si9	0.600	0.602	—
qwl1	0.497	0.498	—
qwl2	0.606	0.610	—
qwl3	0.638	0.640	—
qwl4	0.622	0.623	—
qwl5	0.685	0.687	—
qwl6	0.428	0.425	—
qwl7	0.694	0.693	—
qwl8	0.858	0.857	—
qwl9	0.727	0.727	—
qwl10	0.398	0.398	—
qwl11	0.473	0.470	—
qwl12	0.413	0.413	—
qwl13	0.446	0.444	—

Note. Six QWL loadings are below 0.500 in the five-factor candidate. The complete models fit inadequately; loadings do not validate these constructs.

Table S3. Local PI candidate fit.

Candidate	Scaled χ^2 (df)	Robust CFI	Robust TLI	Robust RMSEA	RMSEA 90% CI	SRMR
PI one-factor	62.76 (9)	0.936	0.894	0.153	[0.114, 0.194]	0.060
PI two-factor	11.65 (8)	0.989	0.979	0.067	[0.000, 0.120]	0.023

Note. Local fit is not global measurement adequacy. The PI-only two-factor comparison has only eight degrees of freedom and a wide robust RMSEA interval. The split was post hoc.

Numerical identification diagnostics: the full-sample derivative matrices have ranks/columns of 30/30 (PI one-factor), 31/31 (PI two-factor), 136/136 (complete four-factor), and 140/140 (complete five-factor). Each candidate converged and passed lavaan post-checks. The covariance matrix of the parameter estimates had a positive minimum eigenvalue, and the residual-variance entries of the unstandardized theta matrix were nonnegative. In these single-group ordinal theta models, the item residual variances are fixed at 1 for scale identification; their non-negativity is therefore not an independent model-admissibility check. These are local numerical diagnostics, not proof of global identification, stable dimensionality, or content validity. The same diagnostic output is supplied for both reduced samples.

In the unchanged historical diagnostic output, vcov_min_eigenvalue refers to the covariance matrix of the parameter estimates returned by lavInspect(fit, 'vcov'). The legacy field negative_standardized_residual_variances counts negative diagonal elements of lavInspect(fit, 'est')$theta; despite its historical name, it is not a check of standardized-solution residual variances. The code and original output labels have been preserved, not rewritten as a new run.

Table S4. Underlying-response correlation residual summaries.

Candidate	Pairs	RMS residual	Max |residual|	|residual| > 0.100	|residual| > 0.200
PI-only one-factor	15	0.071	0.210	2	1
PI-only two-factor	15	0.028	0.056	0	0
Full four-factor	325	0.087	0.268	84	8
Full five-factor	325	0.078	0.266	66	5

Note. These are off-diagonal correlation residual diagnostics, not standard-normal residual tests. No residual covariances were freed on their basis.

S3. Sensitivity analyses

Table S5. Robust complete-model fit across retained samples.

Sample (n)	Candidate	CFI	TLI	RMSEA	RMSEA 90% CI	SRMR
Full (237)	four-factor	0.728	0.698	0.118	[0.111, 0.125]	0.083
Full (237)	five-factor	0.775	0.747	0.108	[0.101, 0.115]	0.075
Record-reduced (229)	four-factor	0.748	0.720	0.111	[0.104, 0.119]	0.079
Record-reduced (229)	five-factor	0.791	0.765	0.102	[0.095, 0.110]	0.071
Pattern-screened (226)	four-factor	0.752	0.725	0.110	[0.103, 0.118]	0.078
Pattern-screened (226)	five-factor	0.794	0.769	0.101	[0.094, 0.109]	0.071

Note. Neither sample reduction resolves complete-model misfit. Composite-score regression likewise does not repair the poor complete-model fit or the low QWL AVE = 0.351 reported in main Table 4.

Table S6. Primary observed-score regression sensitivity.

Sample (n)	b	HC3 SE	95% HC3 CI	β	R^2
Full (237)	-0.307	0.062	[-0.430, -0.185]	-0.396	0.300
Record-reduced (229)	-0.303	0.065	[-0.430, -0.177]	-0.390	0.298
Pattern-screened (226)	-0.314	0.065	[-0.441, -0.188]	-0.404	0.307

Note. Same covariate set in all samples. Primary N = 237. Full component estimates and all standardized products are available in CSV and main Tables 6–7.

Omitting the retained five-item social-isolation index gives b = -0.381, HC3 95% CI [-0.474, -0.288]. Quadratic PI coefficient = 0.056, p = 0.221; Huber PI coefficient = -0.274. Thirteen cases exceed Cook's D > 4/n. No cases were removed from the primary sample on that basis. VIFs for PI and SI are 1.821 and 1.748; some age/tenure dummy VIFs are higher, up to 9.116, which cautions against precise interpretation of demographic coefficients. The complete VIF list is in association_results.json. No quadratic substantive claim is retained.

The resampling unit was the individual retained record. Recruitment-location and WeChat-group identifiers were not retained, so dependence within recruitment sources could not be assessed or modeled. HC3 and record-level bootstrap intervals do not adjust for such clustering.

S4. Revision-stage exploratory factor analysis

All six PI items and all 237 records were retained. A polychoric matrix was estimated; its minimum eigenvalue was 0.219. MINRES common-factor extraction and oblimin rotation were used. Parallel analysis used 1,000 null datasets formed by independent within-item resampling, seed 20260831, a 95th-percentile reference, sim = FALSE, SMC = FALSE, correct = 0.5, and psych::fa.parallel with fa = both and fm = minres. The factor reference uses one-factor communalities rather than PCA eigenvalues. Common factors and components are different targets; both references are reported.

Table S7a. Parallel-analysis retention diagnostics.

Rank	Common-factor eigenvalue	Null factor 95th	Factor retained	PCA eigenvalue	Null PCA 95th
1	3.404	1.061	Yes	3.800	1.345
2	0.288	0.223	Yes	0.858	1.196
3	0.017	0.121	No	0.454	1.088
4	-0.007	0.042	No	0.367	1.005
5	-0.050	-0.025	No	0.301	0.927
6	-0.247	-0.095	No	0.219	0.843

Note. Two common factors exceed their references; only one component exceeds its PCA reference. These are post-hoc diagnostics on the same sample.

Table S7b. One- and two-factor MINRES pattern loadings.

Item	One-factor	Two-factor F1	Two-factor F2	h^2 (two)	u^2 (two)
pi1	0.552	-0.006	1.001	0.997	0.003
pi2	0.679	0.442	0.370	0.495	0.505
pi3	0.820	0.788	0.057	0.668	0.332
pi4	0.891	0.883	0.026	0.803	0.197
pi5	0.798	0.847	-0.047	0.679	0.321
pi6	0.731	0.790	-0.065	0.577	0.423

Note. Signs/orientation are arbitrary. F1 and F2 are not labeled as validated relational/developmental scales. F2 is dominated by pi1; pi2 cross-loads. Oblique pattern coefficients can exceed 1 without a negative uniqueness; nevertheless pi1 uniqueness near zero raises stability concerns.

Table S7c. Variance summaries from the extraction.

Factors	Component of solution	Metric	Value
1	MR1	SS loadings	3.404
1	MR1	Proportion Var	0.567
2	MR1	SS loadings	3.003
2	MR1	Proportion Var	0.501
2	MR2	SS loadings	1.217
2	MR2	Proportion Var	0.203

Note. Two-factor cumulative variance proportion = 0.703 (70.3%). For oblique solutions, factor contributions are not independent causal shares.

Table S7d. Additional exploratory diagnostics.

Diagnostic	Result
KMO	0.854
Approximate Bartlett χ^2 (df)	775.77 (15), $p < 0.001$
Two-factor correlation	0.499
Null parallel-analysis warnings	26 ultra-Heywood warnings; full warning log retained
Independent validation sample	None

Note. Bartlett is only an approximate descriptive check when applied to this polychoric matrix. Warnings occurred during null parallel analysis, not negative-variance findings from the CFA models. The observed pattern still does not cleanly recover the proposed split.

The parallel-analysis factor count is not treated as a validation decision. The second factor has narrow indicator coverage and pi1 is close to zero uniqueness; pi2 does not align cleanly with pi1. The common-factor solution therefore provides a reason for caution, not a basis for promoting the earlier two-/four-item scores into validated constructs. No new scoring scheme was selected from this EFA.

S5. Previously proposed dimensional scores: exploratory associations

Table S8. Joint-score exploratory regressions (N = 237).

Outcome/model	Term	b	HC3 SE	95% HC3 CI	β
Adjusted QWL	dev_c	-0.230	0.064	[-0.356, -0.105]	-0.344
Adjusted QWL	rel_c	-0.110	0.062	[-0.231, 0.012]	-0.155
Career-development constraints	dev_c	0.553	0.100	[0.356, 0.750]	0.457
Career-development constraints	rel_c	0.149	0.096	[-0.039, 0.337]	0.117
Conditional QWL	dev_c	-0.149	0.066	[-0.278, -0.020]	-0.223
Conditional QWL	rel_c	-0.088	0.062	[-0.208, 0.033]	-0.124

Note. dev_c = centered mean(pi1, pi2); rel_c = centered mean(pi3–pi6), after recoding. Both scores enter jointly with the primary covariates. cds_c denotes the perceived career-development constraints score. Conditional QWL models additionally include cds_c.

Table S9. Exploratory bootstrap products and direct dimensional contrasts.

Quantity	Estimate	Bootstrap SE	95% bootstrap CI
dev_adjusted_qwl_b	-0.230	0.062	[-0.352, -0.112]
rel_adjusted_qwl_b	-0.110	0.060	[-0.228, 0.010]
dev_minus_rel_adjusted_qwl_b	-0.121	0.104	[-0.328, 0.078]
dev_minus_rel_adjusted_qwl_beta	-0.189	0.150	[-0.488, 0.102]
dev_indirect_ab	-0.081	0.027	[-0.139, -0.036]
rel_indirect_ab	-0.022	0.016	[-0.056, 0.006]
dev_minus_rel_indirect_ab	-0.059	0.028	[-0.124, -0.013]
dev_indirect_ab_standardized	-0.122	0.038	[-0.202, -0.055]
rel_indirect_ab_standardized	-0.031	0.022	[-0.080, 0.009]
dev_minus_rel_indirect_ab_standardized	-0.091	0.041	[-0.181, -0.021]

Note. 5,000 case-resampling draws; seed 20260811; percentile intervals; SDs recomputed for standardized quantities. dev_minus_rel compares coefficients/products directly. No multiplicity adjustment; exploratory interpretation only. Scores share a 1–5 response metric, not a validated common latent unit.

The adjusted QWL coefficient difference (developmental minus relational) is -0.121, 95% bootstrap CI [-0.328, 0.078]; this does not support a difference in those adjusted coefficients. The indirect-product difference is -0.059, 95% CI [-0.124, -0.013]. The latter is an exploratory statistical contrast with overlapping content and unstable dimensional evidence, not a validated difference in causal mechanisms. All contrasts are retained, including intervals containing zero. A significant coefficient for one score and a nonsignificant coefficient for the other is not itself a between-score test.

S6. Alternative summaries, software and provenance

Table S10. Four-factor HTMT2 matrix.

Factor	1	2	3	4
1. Professional isolation	—	—	—	—
2. Perceived career-development constraints	0.593	—	—	—
3. Social-isolation index	0.734	0.489	—	—
4. QWL	0.460	0.553	0.395	—

Note. Absolute polychoric, geometric means. Does not override poor fit.

Table S11. Fit-index variants without selective replacement.

Candidate	Variant	CFI	TLI	RMSEA
PI-only one-factor	Standard	0.994	0.989	0.099
PI-only one-factor	Scaled	0.973	0.956	0.159
PI-only one-factor	Robust	0.936	0.894	0.153
PI-only two-factor	Standard	1.000	1.002	0.000
PI-only two-factor	Scaled	0.998	0.997	0.044
PI-only two-factor	Robust	0.989	0.979	0.067
Full four-factor	Standard	0.961	0.957	0.096
Full four-factor	Scaled	0.900	0.889	0.086
Full four-factor	Robust	0.728	0.698	0.118
Full five-factor	Standard	0.971	0.968	0.083
Full five-factor	Scaled	0.920	0.910	0.077
Full five-factor	Robust	0.775	0.747	0.108

Note. The manuscript uses robust categorical fit indices for interpretation. Standard/scaled values are disclosed for transparency and are not substituted to improve apparent fit.

Software used for the retained v8 reanalysis: Python 3.12.13; numpy 2.3.5; pandas 3.0.1; scipy 1.18.1; statsmodels 0.15.0; pyreadstat 1.3.6. R 4.6.1; lavaan 0.7-2; semTools 0.5-9; psych 2.6.5; GPArotation 2026.8-2; foreign 0.8-91; jsonlite 2.0.0. Python performs score preparation, OLS/HC3, regression diagnostics and case-resampling bootstrap; R performs ordinal CFA, HTMT2 and polychoric EFA/parallel analysis.

The archived RUN_MANIFEST_v8.json identifies the analysis scripts and aggregate outputs by SHA256. The accompanying SYNC_MANIFEST_v9_5.json records their unchanged hashes and current relative paths; it is not a new analysis-run record. The analysis source is Raw Dataset.sav, SHA256 95aa7932e5cf953e19c40cea557b028a18521a383eff875c373313b51814145c. The participant-level source data, participant-level CSVs and R fit-object archive are not distributed with these supporting files. Reproduction requires authorized access to the source and the project structure described in reproducibility/README.md. Software availability/version differences may affect reproducibility. The complete variable dictionary is in reproducibility/codebook/codebook.md, with CSV and JSON versions in the same directory. These documentation files are new; the archived statistical scripts and outputs are unchanged.

Access to analysis code and statistical outputs does not authorize redistribution of participant-level data. As stated in the main Data availability statement, de-identified participant-level data are not public because the retained participant information did not explicitly authorize unrestricted public redistribution. Qualified researchers may contact Rungang Liu (rgliu@cczu.edu.cn); access is subject to ethics/institutional approval and data-protection requirements. Questionnaire documentation remains subject to applicable permissions.

Author-confirmed ethics and consent: The study involving human participants was reviewed and approved by the Institutional Review Board/Ethics Committee of the Qu Qiubai School of Government, Changzhou University (Protocol No. CZU-SG-2025-010; decision dated 25 May 2025). Data collection was conducted after this approval. Informed consent was obtained through a questionnaire-embedded checkbox or confirmation item indicating that participants had read the information and agreed to participate. Individuals could decline or discontinue participation if they did not wish to participate or considered that their circumstances did not meet the survey requirements. The approval certificate records voluntary informed consent before participation. The authors confirmed that collection followed approval, but exact data-collection dates were not retained. See main Section 2.2 and the Ethics statement.

Author contributions: Xingqiang Xu: Conceptualization, Data curation, Investigation, Methodology, Writing – original draft. Rungang Liu: Formal analysis, Supervision, Validation, Writing – review and editing. Both authors reviewed and approved the revised manuscript. These reported contributions have been confirmed by the authors; the main Author contributions statement is unchanged.

AI-use disclosure: Generative AI was used in the preparation and revision of this manuscript. ChatGPT (GPT-5.5 for the original manuscript; GPT-5.6 for earlier revisions; OpenAI; https://chatgpt.com/) and OpenAI Codex (OpenAI; https://openai.com/codex/) assisted with coding and code review, source and reference-format checks, manuscript organization, English-language editing, and document preparation during July–September 2026. OpenAI Codex (GPT-6) also assisted with the September 2026 author-side review, reporting revision, and variable-codebook preparation. These tools did not generate participant responses. Author-directed data correction and deterministic recoding are disclosed in Sections 2.3 and 2.5. The authors reviewed and approved the revised manuscript and are responsible for its content. See main Section 2.7 and the Generative AI statement.

Remaining archival limitations are unchanged: exact collection dates, a separately recorded minimum-age criterion, respondent-level occupational-screening records, the original excluded questionnaires and their case-level exclusion reasons were not preserved. Administered-questionnaire equivalence, full scale-adaptation records and the selection rationale for the five-item SI index remain unverified or unavailable. The confirmations above do not resolve these gaps or validate the measurement model. Recruitment-cluster identifiers were also unavailable. The retained job-level labels do not verify occupational classification or eligibility, and neither numerical identification nor same-sample dimensional analysis establishes a stable two-dimensional structure.
